# U-shaped association between sleep duration and urinary albumin excretion in Korean adults: 2011-2014 Korea National Health and Nutrition Examination Survey

**DOI:** 10.1371/journal.pone.0192980

**Published:** 2018-02-22

**Authors:** Ji Hee Yu, Kyungdo Han, Nam Hoon Kim, Hye Jin Yoo, Ji A. Seo, Sin Gon Kim, Kyung Mook Choi, Sei Hyun Baik, Nan Hee Kim

**Affiliations:** 1 Division of Endocrinology and Metabolism, Department of Internal Medicine, Korea University College of Medicine, Ansan, Korea; 2 Department of Biostatics, College of Medicine, The Catholic University of Korea, Seoul, Korea; Graduate School of Medicine, University of the Ryukyus, JAPAN

## Abstract

Although sleep duration has been extensively studied in metabolic diseases, few studies have investigated the impact of sleep duration on chronic kidney disease. The aim of this study was to examine the relationship between sleep duration and albuminuria in the general population. Among 24,948 adults who participated in the 2011–2014 KNHANES, a total of 19,994 subjects were included in this analysis. Subjects were categorized into the following five groups according to self-reported sleep duration: less than 5 h, 6 h, 7 h, 8 h, and more than 9 h. The association between sleep duration and urinary albumin-creatinine ratio (UACR) was examined cross-sectionally. Subjects with both short and long sleep durations were significantly associated with higher UACR levels and higher proportions of patients with microalbuminuria (30–299 mg/g) and macroalbuminuria (≥300 mg/g) compared to those with a sleep duration of 7 hours. The U-shaped association between sleep duration and UACR remained significant even after adjustment for potential confounders, including age, sex, body mass index, smoking, alcohol, education, income, exercise, estimated glomerular filtration rate, diabetes mellitus, hypertension and hypercholesterolemia. The U-shaped association is more evident in the subgroup aged 65 or older, or in female subjects. Our findings suggest that both short and long sleep durations have a U-shaped association with UACR levels in the general population, independent of potential confounders.

## Introduction

Chronic kidney disease (CKD) is a growing public health problem that afflicts an estimated 13% of individuals worldwide [1]. CKD is a major risk factor for end-stage renal disease, cardiovascular disease, and premature death [2]. Therefore, CKD prevention and management continue to be major public health challenges. The Kidney Disease Improving Global Outcomes (KDIGO) guidelines define and stage CKD by glomerular filtration rate (GFR) and albuminuria, which are considered markers of kidney damage [3]. An elevated urinary albumin excretion is independently predictive for development of renal function impairment in the general population [4].

Adequate sleep duration is crucial for regulation of body metabolism and quality of life. Both short and long sleep durations are associated with adverse health outcomes, including obesity [5], type 2 diabetes [6], hypertension [7] and cardiovascular disease [8]. Short and long sleep durations were also associated with increased risk of atherosclerosis, as measured by the intima-media thickness of the common carotid arteries [9]. Furthermore, short and long sleep durations are associated with increased mortality [10].

Although sleep disruption has been studied extensively in cardiovascular and metabolic disease, epidemiological evidence of associations between sleep duration and chronic kidney disease (CKD) is insufficient. Previous studies have demonstrated an increased risk of CKD or proteinuria in short sleepers [11,12]. A short sleep duration of 6 h or less was significantly related to increased risk of reduced GFR in a Chinese population with hypertension [12]. A recent meta-analysis also showed the potential association between short sleep duration and proteinuria, with an overall 1.47-fold increased risk of proteinuria [11]. However, studies have yet to identify whether these relationships exist in long sleepers. Moreover, there are few studies assessing the relationship between sleep duration and albuminuria, a more sensitive marker of CKD than proteinuria. Although sleep duration was associated with albuminuria in a Japanese study of patients with type 2 diabetes [13,14], few epidemiologic studies have examined the association between sleep duration and albuminuria among the general population. Therefore, the aim of this study was to investigate the association between sleep duration and urinary albumin excretion in Korean adults based on data from the Korean National Health and Nutrition Examination Survey (KNHANES).

## Methods

### Study subjects

This study was based on data from the 2011–2014 KNHANES, a cross-sectional nationally representative survey conducted by the Korean Center for Disease Control and Prevention. The KNHANES consists of a health interview survey, nutrition survey, and health examination survey [15]. The health interview survey is performed using self-administered structured questionnaires to obtain information on sociodemographic characteristics, health status, health service use, and health behaviors. Trained interviewers visit each household and assist participants with specific items in the self-administered tool.

Of 32,144 people who participated in the 2011–2014 survey, 19,994 adults aged ≥19 years were included in the analysis after excluding 7,196 subjects aged <19 years and 4,954 subjects missing information about sleep duration or urinary albumin excretion levels. All participants provided written informed consent, and the Institutional Review Board of the Korean Center for Disease Control and Prevention approved the study protocol for the survey.

### Measurements

#### Sleep duration

Participants filled in a self-reported questionnaire with the question: "How many hours, on average, do you sleep a day?" Based on the answer, subjects were classified into five groups: ≤5, 6, 7, 8, and ≥9 hours. Those reporting 7 h per night served as a reference group.

#### Microalbuminuria

The serum and urinary creatinine concentrations of a random sample were measured using a colorimetric method (Hitachi Automatic Analyzer, Hitachi, Japan). Urinary albumin was measured using a turbidimetric immunoassay (Hitachi Automatic Analyzer 7600, Hitachi, Japan). The UACR (mg/g) levels were calculated by dividing the urinary albumin values by the urinary creatinine concentrations. Albuminuria was defined as UACR ≥30 mg/g, and macroalbuminuria was defined as UACR ≥300 mg/g [16]. The estimated glomerular filtration rate (eGFR) was determined using the Modification of Diet in Renal Disease (MDRD) formula: eGFR (ml/min/1.73m^2^) = 175 x SCr (mg/dl)^-1.154^ x Age^-0.203^ x 0.742 (if female) [17]. Urine albumin and creatinine concentrations were measured in the same laboratory during all surveys. The inter-assay coefficient of variation for all laboratory work was constantly low (<3.1%). The results of detailed health interviews, physical examinations, and other laboratory procedures have been published elsewhere.

#### Covariates

Covariates included in the analyses were age, sex, education, income, current smoking, alcohol consumption, physical activity, body mass index (BMI), waist circumference, serum lipid profiles, hypertension, diabetes, and hypercholesterolemia. Regular exercise was defined as five or more exercise sessions per week (≥30 min/session). Alcohol consumption was assessed by questioning subjects about their drinking behavior, including the average amount of drinking and drinking frequency during the month prior to the interview. The average amount of daily alcohol intake was calculated and categorized into 3 groups: 0 g/day, <30 g/day, and ≥30 g/day. Hypertension was defined as average blood pressure ≥140/90 mmHg or use of antihypertensive medication. Diabetes mellitus was defined as hemoglobin A1c levels ≥6.5%, fasting plasma glucose (FPG) levels ≥7.0 mmol/L, current use of antidiabetic medication, or previous diagnosis of diabetes by a physician [18]. Height and weight were obtained using standardized techniques and equipment. Height was measured to the nearest 0.1 cm using a portable stadiometer (Seriter, Bismarck, ND). Weight was measured to the nearest 0.1 kg using a Giant-150N calibrated balance-beam scale (Hana, Seoul, Korea). BMI was calculated by dividing the weight by the square of the height (kg/m^2^). Systolic and diastolic blood pressure was measured by standard methods using a sphygmomanometer while the patient was seated. Three measurements were recorded for all subjects at 5-min intervals, and the average of the second and third measurements was used in the analysis.

Blood samples were taken after participants fasted for at least eight hours. The samples were immediately centrifuged, refrigerated, and then transported in cold storage to the Central Testing Institute in Seoul, Korea. Samples were analyzed within 24 hours. The serum levels of total cholesterol, high-density lipoprotein (HDL) cholesterol, and triglyceride were measured using a Hitachi Automatic Analyzer 7600 (Hitachi, Tokyo, Japan). The low-density lipoprotein (LDL) cholesterol level was calculated by the Friedewald formula for subjects with serum triglyceride levels <400 mg/dL, and it was measured directly by commercially available kits (Cholestest^®^ LDL; Sekisui Medical, Tokyo, Japan) when triglyceride levels were ≥400 mg/dL. According to the Adult Treatment Panel III guidelines of the National Cholesterol Education Program [19], hypercholesterolemia was defined as total cholesterol level ≥240 mg/dL or current use of lipid-lowering drugs.

### Statistical analysis

The KNHANES participants were not equally representative of the Korean population. Thus, the SAS survey procedure was applied to reflect the complex sampling design and the sampling weights of KNHANES and to provide nationally representative prevalence estimates. After participants were divided into five groups based on sleep duration, analysis of variance and Chi-squared tests with Bonferroni *post hoc* test were performed to determine differences in variables. Multivariate-adjusted UACR levels according to sleep duration were compared using analysis of covariance. Multiple logistic regression analyses were performed to determine the associations between sleep duration and albuminuria after controlling for other covariates (age, sex, BMI, smoking, alcohol, education, income, exercise, eGFR, DM, HTN and hypercholesterolemia). The adjusted odds ratios (ORs) and corresponding 95% confidence intervals (CIs) were obtained to relate each category of sleep duration to albuminuria. The UACR values were log-transformed for statistical analyses. The proportions of patients with microalbuminuria or macroalbuminuria were compared using a Chi-squared test. A *P*-value of <0.05 was considered statistically significant. All statistical analyses were performed using the SAS software package version 9.3 (SAS Institute Inc., Cary, NC).

## Results

Table 1 shows the characteristics of study subjects stratified by sleep duration. Of 19,994 subjects, 3,264 (16.3%) had a sleep duration less than 5 hours, and 1,398 (7.0%) had a sleep duration more than 9 hours. The proportion of female subjects increased in both short (≤5 h) and long (≥9 h) sleepers. eGFR levels were higher, and BMI and waist circumferences were lower in subjects who slept longer in association with their younger age, despite the lower rate of regular exercise. The proportion of subjects with lower income or less education was high in both short and long sleepers. Fasting plasma glucose levels and the rate of patients with diabetes mellitus or hypercholesterolemia increased in both short and long sleepers. Blood pressure and proportion of hypertension were highest in the short sleepers.

**Table 1:** Clinical characteristics of study subjects according to sleep duration.

	Sleep duration (h)					
	≤5	6	7	8	≥9	*P*
n	3264	5450	5555	4327	1398	
Age (years)	52.5 ± 0.4	45.7 ± 0.3	44.9 ± 0.3	43.9 ± 0.3	44.5 ± 0.7	<0.001
Sex, male (%)	42.7 (1.0)	55.1 (0.7)	54.3 (0.7)	52.8 (0.9)	43.4 (1.8)	<0.001
Current smoker (%)	20.8 (0.9)	23.9 (0.8)	22.5 (0.7)	22.9 (0.8)	24.2 (1.6)	0.144
Alcohol intake (≥30 g/day)	10.1 (0.7)	10.6 (0.6)	8.8 (0.5)	9.9 (0.6)	9.5 (1.2)	0.193
Education (≥high school)	54.4 (1.2)	75.3 (0.8)	77.4 (0.8)	75.9 (0.8)	66.7 (1.5)	<0.001
Income (the lowest quartile)	24.7 (1)	12.5 (0.6)	11.5 (0.6)	13.5 (0.7)	22.9 (1.5)	<0.001
Regular exercise (%)	38.8 (1.1)	41.1 (0.8)	39 (0.8)	37.8 (1.0)	32.1 (1.6)	<0.001
DM (%)	13.8 (0.7)	10.1 (0.5)	9.7 (0.5)	10.0 (0.5)	14.5 (1.1)	<0.001
HTN (%)	33.7 (1.1)	25.1 (0.7)	24.4 (0.7)	23.4 (0.8)	25.3 (1.4)	<0.001
Hypercholesterolemia (%)	16.1 (0.8)	12.9 (0.5)	13.4 (0.5)	12.0 (0.6)	13.5 (1.1)	<0.001
BMI (kg/m^2^)	24.0 ± 0.1	23.9 ± 0.1	23.7 ± 0.1	23.6 ± 0.1	23.4 ± 0.1	<0.001
Waist circumference (cm)	82.0 ± 0.2	81.4 ± 0.2	80.9 ± 0.2	80.8 ± 0.2	80.4 ± 0.4	<0.001
SBP (mmHg)	120.2 ± 0.4	117.1 ± 0.3	116.7 ± 0.3	116.5 ± 0.3	116.2 ± 0.6	<0.001
DBP (mmHg)	75.8 ± 0.2	76.1 ± 0.2	75.9 ± 0.2	75.5 ± 0.2	73.8 ± 0.4	<0.001
FPG (mmol/L)	5.50 ± 0.03	5.42 ± 0.02	5.38 ± 0.02	5.40 ± 0.02	5.48 ± 0.04	<0.001
Triglyceride (mmol/L)*	1.30 (1.26–1.33)	1.25 (1.23–1.28)	1.24 (1.22–1.27)	1.26 (1.23–1.29)	1.25 (1.19–1.31)	0.180
Total cholesterol (mmol/L)	4.92 ± 0.02	4.89 ± 0.02	4.89 ± 0.02	4.86 ± 0.02	4.83 ± 0.04	0.096
HDL-cholesterol (mmol/L)	1.36 ± 0.01	1.35 ± 0.01	1.36 ± 0.01	1.35 ± 0.01	1.34 ± 0.01	0.845
LDL-cholesterol (mmol/L)	2.88 ± 0.02	2.87 ± 0.01	2.86 ± 0.01	2.83 ± 0.02	2.80 ± 0.03	0.055
GFR (ml/min/1.73m^2^)	92.8 ± 0.4	94.5 ± 0.3	94.9 ± 0.3	96.4 ± 0.4	98.2 ± 0.7	<0.001
UACR* (mg/g)	6.8 (6.4–7.1)	5.4 (5.2–5.6)	5.2 (5.0–5.4)	5.4 (5.2–5.7)	6.4 (5.9–6.9)	<0.001

Data are presented as % (SE), mean ± SE, or *geometric mean (95% confidence interval (CI)).

*Statistical significance was estimated after logarithmic transformation.

BMI, body mass index; DBP, diastolic blood pressure; DM, diabetes mellitus; FPG, fasting plasma glucose; GFR, glomerular filtration rate; HDL, high-density lipoprotein; HTN, hypertension; LDL, low-density lipoprotein; SBP, systolic blood pressure; UACR, urinary albumin-creatinine ratio.

Table 2 shows adjusted UACR levels according to sleep duration. Both subjects with short and long sleep durations had higher UACR levels compared with those who slept 7 h (*P* <0.001), indicating the presence of association in a U-shaped fashion. These relationships were unchanged even after adjustment for age, sex, BMI, smoking, alcohol, regular exercise, education, income, eGFR, DM, HTN and hypercholesterolemia (*P* <0.001). Conversely, the association of eGFR with sleep duration was significant only in women after adjusting for confounding variables (*P* = 0.024, S1 Table).

**Table 2:** Adjusted urinary albumin-creatinine ratio (UACR) levels according to sleep duration.

	Sleep duration (h)					
	≤5	6	7	8	≥9	*P*
Model 1	6.52 (6.21–6.85)	5.92 (5.69–6.16)	5.77 (5.56–5.98)	6.09 (5.85–6.35)	6.91 (6.40–7.47)	<0.001
Model 2	6.47 (6.16–6.80)	5.89 (5.67–6.12)	5.77 (5.56–5.98)	6.11 (5.87–6.36)	6.94 (6.42–7.50)	<0.001
Model 3	6.35 (6.04–6.66)	5.92 (5.70–6.14)	5.79 (5.59–6.00)	6.10 (5.86–6.35)	6.80 (6.29–7.35)	<0.001
Model 4	6.40 (6.10–6.71)	5.96 (5.75–6.18)	5.80 (5.60–6.01)	6.06 (5.83–6.31)	6.53 (6.06–7.04)	<0.001

Data are presented as geometric mean (95% CI).Model 1: Adjusted for age and sex.

Model 2: Adjusted for age, sex and BMI.

Model 3: Adjusted for age, sex, BMI, smoking, alcohol, education, income and exercise.

Model 4: Adjusted for age, sex, BMI, smoking, alcohol, education, income, exercise, eGFR, DM, HTN and hypercholesterolemia.

**Fig 1:**
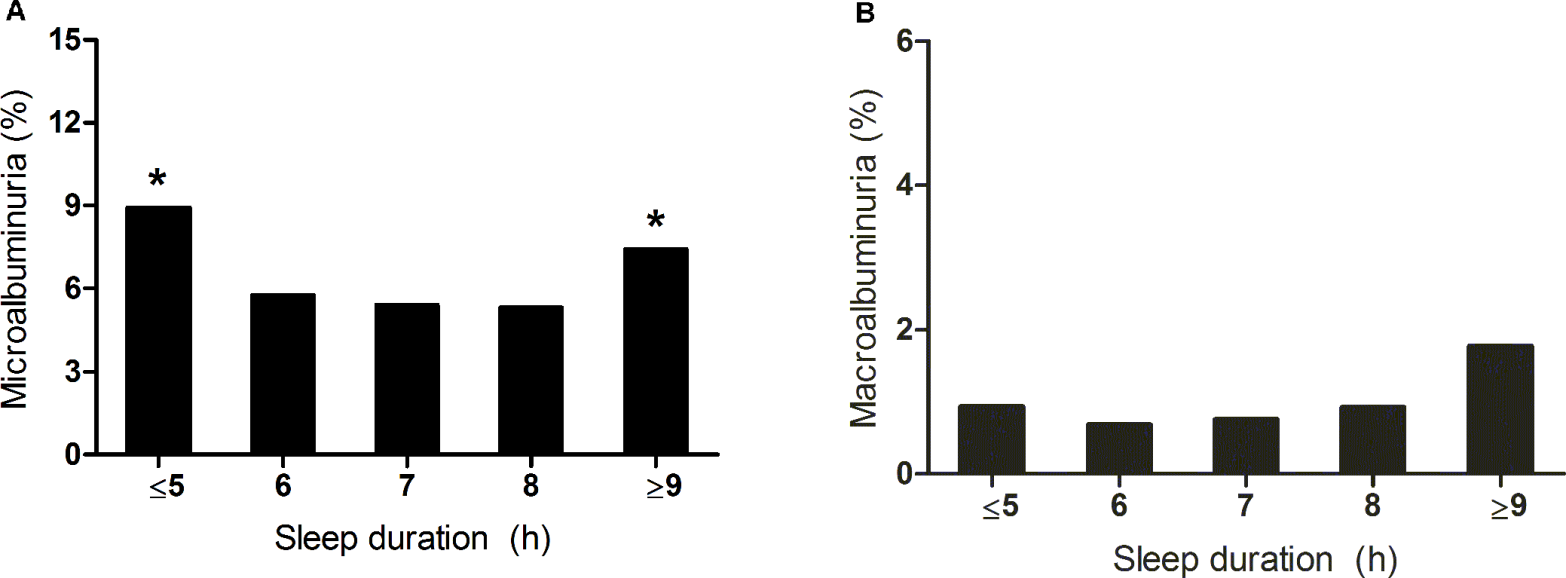
**Proportion of patients with microalbuminuria (A) or macroalbuminuria (B) according to sleep duration.** Microalbuminuria was defined as 30mg/g≤ urinary albumin-creatinine ratio (UACR) <300 mg/g, and macroalbuminuria was defined as UACR ≥300 mg/g. *P <0.005 vs. sleep duration of 7 hours per day.

Fig 1 shows the proportion of subjects with microalbuminuria and macroalbuminuria according to sleep duration. There was also a U-shaped association between sleep duration and the proportion of subjects with albuminuria (*P* <0.001). The proportion of patients with microalbuminuria was 8.9%, 5.7%, 5.4%, 5.3%, and 7.4% among groups with sleep duration of less than 5 h, 6 h, 7 h, 8 h, and more than 9 h, respectively (*P* <0.001, Fig 1A). The proportion of subjects with macroalbuminuria was 0.9%, 0.7% 0.8% 0.9% and 1.8%, respectively (*P* = 0.027, Fig 1B).

Subgroup analyses are shown in Table 3. A significant interaction was observed for gender (interaction *P* = 0.01) and a tendency for age (interaction *P* = 0.10). In all participants, the OR for albuminuria in subjects with sleep duration more than 9 h was 1.36 (95% CI 1.03–1.80) compared with those who slept 7 h. Especially in subjects aged 65 or older, the U-shaped association between sleep duration and albuminuria was obvious. In female subjects, the ORs for albuminuria were 1.47 (1.03–2.10) and 1.32 (1.01–1.73) for subjects with sleep duration more than 9 h and with sleep duration 6 h, respectively, compared to those who slept 7 h. In patients with diabetes mellitus, subjects who slept more than 9 h had increased risk of albuminuria (OR = 1.41; 95% CI = 1.03 to 1.94).

**Table 3:** Odds ratios (95% Cis) for albuminuria according to sleep duration.

	Sleep duration (h)				
Subgroup	≤5	6	7	8	≥9
Total	1.18 (0.96–1.45)	1.04 (0.85–1.27)	1	1.07 (0.87–1.31)	**1.36 (1.03–1.80)**
Age					
< 65 years	1.15 (0.87–1.52)	0.99 (0.77–1.27)	1	0.96 (0.75–1.24)	1.08 (0.74–1.60)
≥65 years	**1.40 (1.03–1.89)**	1.26 (0.93–1.71)	1	1.35 (0.96–1.89)	**1.85 (1.23–2.77)**
Sex					
Male	1.36 (0.98–1.88)	0.82 (0.61–1.12)	1	0.96 (0.71–1.32)	1.21 (0.81–1.81)
Female	1.20 (0.91–1.57)	**1.32 (1.01–1.73)**	1	1.17 (0.89–1.54)	**1.47 (1.03–2.10)**
DM					
Yes	1.21 (0.94–1.55)	0.98 (0.77–1.25)	1	1.08 (0.84–1.39)	**1.41 (1.03–1.94)**
Non	1.20 (0.84–1.73)	1.20 (0.85–1.70)	1	1.05 (0.73–1.52)	1.26 (0.78–2.03)

Data are presented as OR (95% CI). Albuminuria was defined as urinary albumin-creatinine ratio (UACR) ≥30 mg/g.

## Discussion

The present study demonstrated that both short and long sleep durations are significantly associated with higher UACR levels and higher rates of albuminuria compared to intermediate sleep duration in the general Korean population even after adjusting for several confounding factors. Moreover, this finding was more noticeable in elderly people over the age of 65, females, and subjects with diabetes.

Extremes in sleep duration have been consistently associated with chronic medical conditions [5–8]. Recent studies have shown that sleep duration is associated with kidney function. A previous study reported that short sleep duration and daytime sleepiness was associated with increased 24-h urinary albumin excretion levels in patients with type 2 diabetes [14]. In a Japanese study [13], sleep duration has a U-shaped association with the urinary albumin excretion levels in patients with type 2 diabetes, suggesting that not only short sleep but also long sleep is associated with albuminuria. Two longitudinal studies also found similar results in healthy subjects without diabetes. Yamamoto et al. reported short sleep (<5 h) was related to a 28% increase in the incidence of proteinuria over a median 2.5 years among young to middle-aged Japanese adults with no indicators of kidney dysfunction at baseline [20]. The other longitudinal study has shown that each 1-hour decrease in sleep duration was associated with a 1.5 mL/min per 1.73 m^2^ increase in eGFR over 10 years among US adults [21]. However, few studies have investigated the relationship between sleep duration and kidney function among large-scaled, community-based adults. Our study demonstrated the U-shaped association of sleep duration with albuminuria in general population.

Emerging evidence suggests that sleep deprivation could interfere with normal renal physiology. Reduced sleep duration may lead to sympathetic nervous system stimulation and elevation of evening cortisol levels [22,23]. Other contributing mechanisms may include overactivity of the renin-angiotensin-aldosterone system, endothelial dysfunction, and systemic inflammation [24]. Sleep deprivation may also activate endothelin, a powerful vasoconstrictor involved in the pathogenesis of hypertension [25]. Taken together, these findings indicate that short sleep duration may contribute to elevated CKD risk.

Few studies published to date have demonstrated a possible mechanism mediating the effect of long sleep duration as a cause of poor health outcomes. Low socio-economic status and low levels of physical activity are associated with long duration of sleep [26,27]. In our study, long sleepers also showed lower levels of education, income, and physical activity (Table 1). Conversely, the association of long sleep duration with CKD could be explained by residual confounding and comorbidities [28]. Potential confounders may predispose individuals to both long sleep duration and poor kidney function. Sleep fragmentation and alteration of the immune system were other possible explanations for an association between long sleep duration and poor health outcomes. Several studies have demonstrated that long sleep duration and time in bed are associated with increased sleep fragmentation, daytime sleepiness, and decreased sleep quality [29,30]. Long sleepers were also associated with restless leg syndrome or snoring [28]. Restless leg syndrome may increase the risk of CVD, diabetes, and several metabolic disorders via chronic activation of the sympathetic nervous system [31]. Frequent snoring was associated with adiposity, inflammatory markers, and elevated risk of metabolic syndrome [32]. A recent meta-analysis of 72 studies demonstrated that long sleep duration (>8 h) was associated with higher levels of CRP and IL-6, markers of systemic inflammation [33]. Further research is expected to explore the underlying mechanisms linking long sleep duration and CKD.

In our analysis, sex was a significant factor affecting the relationship between sleep duration and albuminuria (interaction *P* = 0.01, S2 and S3 Tables). There was no significant association between sleep duration and albuminuria among men. It has been reported that the risk associated with changes in sleep duration varies by sex [34,35]. In a recent Korean study [36], long sleep duration (≥9 h/day) was associated with a higher prevalence of CKD only in women. These previous publications may support the notion that sleep deprivation has a worse impact on metabolism in women than in men. However, it cannot be determined in the present study if this is a coincidental finding. A prominent U-shaped association was identified between sleep duration and albuminuria, especially in elderly patients. As CKD is a major risk factor for mortality in the elderly [37], subjects with extremely short or long sleep duration should correct their poor sleep hygiene for better health outcomes. In subjects with diabetes, only long sleepers had significantly increased urinary albumin excretion compared to intermediate sleepers. Although the mechanisms that underlie these differences are still unclear, they provide valuable information on a vulnerable group with abnormal sleep duration.

The major strength of our study was the large nationally representative sample of adult Koreans. Nevertheless, our study had several limitations. First, sleep duration was determined according to a self-reported questionnaire, allowing potential misclassification. In this observational study, many of the baseline covariates used in the analyses were based on self-reported information. Second, a single urine spot sample was used to assess UACR. Third, the causal relationship between sleep duration and albuminuria cannot be confirmed due to the cross-sectional design of the study. Fourth, information about sleep quality was not reflected in this study. Fifth, we could not take into account the use of steroid, angiotensin receptor blocker or angiotensin converting enzyme inhibitors, which may affect the level of albuminuria. Sixth, we didn't exclude cancer patients from this study. However, there was no difference in the significance of the results even when we analyzed after excluding cancer patients. Finally, we have not obtained information regarding renal diseases due to nondiabetic causes such as chronic glomerulonephritis. The lack of these informations may have made our findings more inaccurate in some extent.

In conclusion, our findings suggest that both short and long sleep duration has a U-shaped association with UACR levels in the general population, especially in elderly people. Individuals with either short or long sleep durations may have higher risk of CKD and CVD than those with adequate sleep duration because CKD is a major risk factor for end-stage renal disease and CVD. Because sleep duration is a modifiable risk factor, clinicians should consider these findings in the prevention and management of CKD.

## Supporting information

S1 Table The adjusted eGFR levels according to sleep duration. (DOC)

S2 Table Association between urinary albumin-creatinine ratio (UACR) levels and sleep duration according to age and gender. (DOC)

S3 Table Proportion of patients with micoalbuminuria and macroalbuminuria according to sleep duration. (DOC)
