# Supplementary material for: U-shaped association between sleep duration and urinary albumin excretion in Korean adults: 2011-2014 Korea National Health and Nutrition Examination Survey
Source: PLoS One. 2018 Feb 22;13(2):e0192980. doi: 10.1371/journal.pone.0192980 (PMC5823398; doi:10.1371/journal.pone.0192980)
Supplement: S1 Table — (DOC) [file pone.0192980.s001.doc]

**S1 Table. The adjusted eGFR levels according to sleep duration**

|  |  | Sleep duration (h) | | | | | |
| --- | --- | --- | --- | --- | --- | --- | --- |
|  |  | ≤5 | 6 | 7 | 8 | ≥9 | *P* |
| Total | Model 1 | 93.3 ± 0.4 | 92.4 ± 0.3 | 92.4 ± 0.3 | 93.3 ± 0.3 | 94.5 ± 0.6 | 0.006 |
|  | Model 2 | 93.4 ± 0.4 | 92.5 ± 0.3 | 92.4 ± 0.3 | 93.2 ± 0.3 | 94.4 ± 0.6 | 0.007 |
|  | Model 3 | 93.1 ± 0.4 | 92.5 ± 0.3 | 92.6 ± 0.3 | 93.2 ± 0.3 | 94.0 ± 0.6 | 0.136 |
|  | Model 4 | 93.1 ± 0.4 | 92.5 ± 0.3 | 92.7 ± 0.3 | 93.2 ± 0.3 | 94.1 ± 0.6 | 0.109 |
|  |  |  |  |  |  |  |  |
| Men | Model 1 | 91.4 ± 0.6 | 90.6 ± 0.4 | 89.7 ± 0.4 | 90.6 ± 0.4 | 91.3 ± 0.8 | 0.091 |
|  | Model 2 | 91.4 ± 0.6 | 90.7 ± 0.4 | 89.7 ± 0.4 | 90.5 ± 0.4 | 91.1 ± 0.8 | 0.098 |
|  | Model 3 | 91.1 ± 0.5 | 90.7 ± 0.4 | 90.0 ± 0.4 | 90.4 ± 0.4 | 90.5 ± 0.8 | 0.547 |
|  | Model 4 | 91.1 ± 0.5 | 90.8 ± 0.4 | 90.2 ± 0.4 | 90.5 ± 0.4 | 90.8 ± 0.8 | 0.658 |
|  |  |  |  |  |  |  |  |
| Women | Model 1 | 94.9 ± 0.5 | 93.4 ± 0.4 | 94.4 ± 0.4 | 95.1 ± 0.4 | 96.3 ± 0.9 | 0.005 |
|  | Model 2 | 94.8 ± 0.5 | 93.4 ± 0.4 | 94.4 ± 0.4 | 95.1 ± 0.4 | 96.2 ± 0.9 | 0.005 |
|  | Model 3 | 94.7 ± 0.5 | 93.5 ± 0.4 | 94.5 ± 0.4 | 95.0 ± 0.4 | 95.9 ± 0.9 | 0.027 |
|  | Model 4 | 95.0 ± 0.5 | 93.7 ± 0.4 | 94.7 ± 0.4 | 95.3 ± 0.4 | 96.2 ± 0.9 | 0.024 |

Data presented as geometric mean (95% CI).
Model 1 : Adjusted for age and sex.

Model 2: Adjusted for age, sex and BMI.

Model 3: Adjuested for age, sex, BMI, smoke, drink, education, income and exercise

Model 4: Adjusted for age, sex, BMI, smoke, drink, education, income, exercise, DM, HTN, Hyperlipidemia
