# Supplementary material for: U-shaped association between sleep duration and urinary albumin excretion in Korean adults: 2011-2014 Korea National Health and Nutrition Examination Survey
Source: PLoS One. 2018 Feb 22;13(2):e0192980. doi: 10.1371/journal.pone.0192980 (PMC5823398; doi:10.1371/journal.pone.0192980)
Supplement: S2 Table — (DOC) [file pone.0192980.s002.doc]

**S2 Table. Association between urinary albumin-creatinine ratio (UACR) levels and sleep duration according to age and gender**

|  | Sleep duration (h) | | | | | |
| --- | --- | --- | --- | --- | --- | --- |
|  | ≤5 | 6 | 7 | 8 | ≥9 | *P* |
| Age <65 |  | | | | | |
| Model 1 | 5.64 (5.34-5.96) | 5.26 (5.05-5.47) | 5.15 (4.96-5.34) | 5.36 (5.14-5.59) | 5.84 (5.36-6.36) | 0.005 |
| Model 2 | 5.59 (5.29-5.9) | 5.24 (5.04-5.44) | 5.16 (4.97-5.35) | 5.37 (5.15-5.6) | 5.85 (5.37-6.37) | 0.007 |
| Model 3 | 5.51 (5.21-5.82) | 5.23 (5.03-5.44) | 5.17 (4.98-5.37) | 5.34 (5.12-5.57) | 5.72 (5.25-6.23) | 0.062 |
| Model 4 | 5.60 (5.30-5.91) | 5.24 (5.05-5.44) | 5.14 (4.95-5.33) | 5.24 (5.02-5.46) | 5.46 (5.02-5.94) | 0.056 |
| Age ≥65 |  | | | | | |
| Model 1 | 10.41 (9.57-11.32) | 9.55 (8.71-10.46) | 9.04 (8.24-9.92) | 10.25 (9.21-11.4) | 12.74 (10.94-14.83) | 0.004 |
| Model 2 | 10.41 (9.58-11.32) | 9.53 (8.71-10.43) | 9.08 (8.27-9.96) | 10.25 (9.23-11.4) | 12.75 (10.96-14.82) | 0.004 |
| Model 3 | 10.30 (9.46-11.21) | 9.54 (8.69-10.48) | 9.06 (8.23-9.98) | 10.13 (9.10-11.28) | 12.62 (10.83-14.70) | 0.009 |
| Model 4 | 10.09 (9.21-11.05) | 9.51 (8.67-10.43) | 8.59 (7.83-9.43) | 10.24 (9.15-11.46) | 11.39 (9.59-13.52) | 0.020 |
| Male |  |  |  |  |  |  |
| Model 1 | 6.11 (5.64-6.61) | 5.46 (5.20-5.73) | 5.29 (5.04-5.55) | 5.70 (5.38-6.04) | 6.19 (5.52-6.95) | 0.003 |
| Model 2 | 6.10 (5.63-6.60) | 5.41 (5.16-5.68) | 5.28 (5.03-5.54) | 5.72 (5.40-6.06) | 6.28 (5.60-7.05) | 0.001 |
| Model 3 | 6.01 (5.55-6.5) | 5.42 (5.17-5.69) | 5.29 (5.04-5.55) | 5.70 (5.38-6.03) | 6.17 (5.50-6.92) | 0.007 |
| Model 4 | 6.10 (5.66-6.57) | 5.48 (5.23-5.73) | 5.28 (5.03-5.53) | 5.64 (5.34-5.95) | 5.82 (5.24-6.46) | 0.010 |
| Female |  |  |  |  |  |  |
| Model 1 | 6.92 (6.53-7.33) | 6.34 (6.02-6.67) | 6.20 (5.91-6.50) | 6.39 (6.06-6.73) | 7.50 (6.76-8.31) | 0.001 |
| Model 2 | 6.89 (6.50-7.29) | 6.33 (6.01-6.67) | 6.20 (5.92-6.50) | 6.40 (6.07-6.74) | 7.46 (6.73-8.27) | 0.002 |
| Model 3 | 6.75 (6.37-7.15) | 6.38 (6.06-6.72) | 6.23 (5.94-6.53) | 6.41 (6.08-6.75) | 7.28 (6.57-8.07) | 0.025 |
| Model 4 | 6.80 (6.43-7.19) | 6.41 (6.10-6.74) | 6.26 (5.99-6.55) | 6.39 (6.07-6.72) | 7.07 (6.38-7.82) | 0.054 |

Data presented as geometric mean (95% CI).
Model 1 : Adjusted for age and sex.

Model 2: Adjusted for age, sex and BMI.

Model 3: Adjuested for age, sex, BMI, smoke, drink, education, income and exercise

Model 4: Adjusted for age, sex, BMI, smoke, drink, education, income, exercise, DM, HTN, Hyperlipidemia
