# Supplementary material for: U-shaped association between sleep duration and urinary albumin excretion in Korean adults: 2011-2014 Korea National Health and Nutrition Examination Survey
Source: PLoS One. 2018 Feb 22;13(2):e0192980. doi: 10.1371/journal.pone.0192980 (PMC5823398; doi:10.1371/journal.pone.0192980)
Supplement: S3 Table — (DOC) [file pone.0192980.s003.doc]

**S3 Table. Proportion of patients with micoalbuminuria and macroalbuminuria according to sleep duration**

|  | Sleep duration (h) | | | | |  |
| --- | --- | --- | --- | --- | --- | --- |
|  | ≤5 | 6 | 7 | 8 | ≥9 | *P* |
| Total subjects |  |  |  |  |  |  |
| microalbuminuria(%) | 8.93 | 5.75 | 5.4 | 5.33 | 7.43 | <0.001 |
| macroalbuminuria(%) | 0.94 | 0.69 | 0.76 | 0.93 | 1.78 | 0.027 |
| Age<65 |  |  |  |  |  |  |
| microalbuminuria(%) | 5.82 | 4.69 | 4.60 | 4.19 | 4.27 | <0.001 |
| macroalbuminuria(%) | 0.67 | 0.49 | 0.63 | 0.78 | 1.29 | 0.174 |
| Age≥65 |  |  |  |  |  |  |
| microalbuminuria(%) | 16.85 | 13.37 | 12.32 | 14.02 | 21.05 | <0.001 |
| macroalbuminuria(%) | 1.64 | 2.10 | 1.89 | 2.06 | 3.92 | 0.182 |
| Men |  |  |  |  |  |  |
| microalbuminuria(%) | 8.09 | 4.16 | 5.38 | 5.19 | 8.79 | <0.001 |
| macroalbuminuria(%) | 1.17 | 0.78 | 0.90 | 0.95 | 0.87 | 0.858 |
| Women |  |  |  |  |  |  |
| microalbuminuria(%) | 9.56 | 7.69 | 5.43 | 5.5 | 6.39 | <0.001 |
| macroalbuminuria(%) | 0.77 | 0.57 | 0.60 | 0.90 | 2.48 | <0.001 |

Microalbuminuria was defined as 30 mg/g≤ urinary albumin-creatinine ratio (UACR) <300 mg/g, and macroalbuminuria was defined as UACR ≥300 mg/g.
